# Residual Volume of Lymph Nodes During Chemoradiotherapy Based Nomogram to Predict Survival of Nasopharyngeal Carcinoma Patient Receiving Induction Chemotherapy

**DOI:** 10.3389/fonc.2021.739103

**Published:** 2021-09-06

**Authors:** Yan Li, Jian Zang, Jingyi Liu, Shanquan Luo, Jianhua Wang, Bingxin Hou, Lina Zhao, Mei Shi

**Affiliations:** Department of Radiation Oncology, Xijing Hospital, Fourth Military Medical University, Xi’an, China

**Keywords:** nasopharyngeal carcinoma, tumor volume, induction chemotherapy, adaptive chemoradiotherapy, nomogram

## Abstract

**Purpose:**

To accurately stratify nasopharyngeal carcinoma (NPC) patients who were benefit from induction chemotherapy (IC) followed by chemoradiotherapy (CCRT), we established residual volume of lymph nodes during chemoradiotherapy based nomogram to predict survival for NPC patients.

**Methods:**

Cox regression analysis were used to evaluate predictive effects of tumor volume parameters. Multivariate Cox regression analysis was used to identify the prognostic factors, and nomogram models were developed to predict survival of NPC patients receiving IC followed by CCRT.

**Results:**

Compared with other tumor volumetric parameters, midRT GTVnd was the best predictive factor for OS (HR: 1.043, 95%CI: 1.031-1.055), PFS (HR: 1.040, 95%CI: 1.030- 1.051), and DMFS (HR: 1.046, 95%CI: 1.034 – 1.059) according to the HR of Cox regression analysis. Based on multivariate analysis, three nomograms included midRT GTVnd were constructed to predict 4-year survival. The C-index of nomograms for each survival endpoints were as follow (training cohort *vs*. validation cohort): 0.746 *vs*. 0.731 for OS; 0.747 *vs*. 0.735 for PFS; 0.768 *vs*. 0.729 for DMFS, respectively. AUC showed a good discriminative ability. Calibration curves demonstrated a consistence between actual results and predictions. Decision curve analysis (DCA) showed that the nomograms had better clinical predictive effects than current TNM staging system.

**Conclusion:**

We identified the best volumetric indicator associated with prognosis was the residual volume of lymph nodes at the fourth week of chemoradiotherapy for patients receiving IC followed by CCRT. We developed and validated three nomograms to predict specific probability of 4-year OS, PFS and DMFS for NPC patient receiving IC followed by CCRT.

## Introduction

More than 70% of newly diagnosed NPC are classified as locoregionally advanced disease (1). Based on results of several clinical randomized control studies, induction chemotherapy (IC) followed by concurrent chemoradiotherapy (CCRT) has been recommended as a preferred regimen for locoregionally advanced NPC (LA-NPC) by guideline of National Comprehensive Cancer Network (NCCN) and Chinese Society of Clinical Oncology (CSCO) (2, 3). Unfortunately, approximate 20-30% patients could not benefit from IC-CCRT regime, and the toxicities were increased compared with CCRT (4, 5). Therefore, it’s important to identify the patients who could benefit from IC followed by CCRT (4–8).

Emerging evidences show that pretreatment tumor volume is a prognostic factor for disease progression and survival of NPC (9–11). Recent study reported that post IC primary gross tumor and lymph node volume also had prognostic value for overall survival (OS) of LA-NPC (12). The changing rate of primary tumor volume before and after IC has also been demonstrated to predict the survival outcome of NPC (13). However, in clinical practice, tumor with poor response to IC could still respond to chemoradiotherapy and residual tumor with good response to IC could resist to chemoradiotherapy. Because adaptive radiotherapy (ART) can compensate for the dosimetric impacts induced by anatomic and geometric variations in patients, it has been widely used to treat head neck cancer (14, 15). Meanwhile, it also provides opportunity to dynamically evaluate the changing of tumor volume during radiotherapy (16). Several studies found changing of primary tumor volume during CCRT or radiotherapy could impact on patient survival in many cancers (17, 18). With regard to NPC, changing rate of total volume during radiotherapy included primary site and lymph nodes was also reported as a better prognostic factor for NPC patients receiving adaptive CCRT (19). Therefore, the tumor volume change related to IC alone was not adequate for outcomes prediction of NPC patients receiving IC followed by CCRT.

To our knowledge, no study has thus far investigated the detailed volumetric parameters and volume change rate before and after IC as well as during radiotherapy. Therefore, the purpose of the present research was to investigate the predictive volumetric parameters in the whole process of IC followed by CCRT treatment, and further to establish the nomogram to stratify LA-NPC patients who could benefit from IC followed by CCRT.

## Materials and Methods

### Patients

We consecutively reviewed 262 LA-NPC patients at the XiJing Hospital between July 2010 and September 2017. All patients had complete history and physical examinations, blood work and direct fiberoptic nasopharyngoscopy, imaged by computed tomography (CT) and magnetic resonance imaging (MRI) of head and neck, and chest images, abdominal sonography, and whole-body bone scan. Patients were re-staged according to the 8th edition of American Joint Committee for Cancer Staging (AJCC) system. Two radiologists reviewed all the imaging records and disagreements were resolved by consensus. The eligibility criteria in the study included: (1) age≥18 years and Karnofsky performance score ≥70; (2) histologically confirmed newly diagnostic nasopharyngeal squamous cell carcinoma; (3) stages III–IV without distant metastasis; (4) receiving IC+CCRT as initial treatment modality; (5) treated with intensity-modulated radiotherapy (IMRT); (5) re-scanning and re-planning were conducted during chemoradiotherapy. The exclusion criteria included: (1) non-squamous cell carcinoma of nasopharynx; (2) not complete the prescribed course of radiotherapy, (3) without adaptive re-planning during radiotherapy course. Ultimately, a total of 253 patients were included for analysis. The protocol was approved by the appropriate ethical review boards of XiJing hospital, and the study was conducted in accordance with the principles of the Declaration of Helsinki.

### Radiation Therapy and Chemotherapy

The treatment planning approaches were described by our previous studies (20–22). In general, patients were immobilized in the supine position with head, neck, and shoulder thermoplastic mask, and CT simulation according to standard procedures. The target of nasopharynx tumor was delineated manually according to MRI before and after chemotherapy and during radiotherapy. For tumor involved cavity, such as nasal cavity, nasopharynx cavity or oropharynx cavity, the delineation would be changed if primary tumor shrunk in these sites after chemotherapy and during radiotherapy. However, the delineation of primary tumor volume was not changed after chemotherapy and during radiotherapy for tumor involved submucosal sites, skull base, cervical vertebra and intracranial extension. The target of lymph node was delineated according to the imaging before and after chemotherapy and during radiotherapy. If changing of lymph node was observed after chemotherapy and during radiotherapy, the target would be modified according to imaging. The prescribed radiation doses were defined as follows: a total of 72.6 Gy in 33 fractions at 2.2 Gy per fraction to the primary tumor of nasopharynx, 66–72.6 Gy to metastatic lymph nodes, 55–60 Gy to high-risk clinical target, and 50 Gy to low-risk clinical target. All patients were treated with 1 fraction daily for 5 days per week. The doses received by each organ at risk (OAR) should be no more than its tolerance (23).

The induction chemotherapy included TP regimen (docetaxel 75mg/m^2^, cisplatin 75mg/m^2^), GP regimen (gemcitabine 1000mg/m^2^, cisplatin 75mg/m^2^) and TPF regimen (docetaxel 75mg/m^2^, cisplatin 75 mg/m^2^, 5-FU 750 mg/m^2^ days1 to 5) every 3 weeks for 2–3 cycles. Radiotherapy began at 3 weeks after the last cycle of induction chemotherapy. Concurrent chemotherapy was only consisted of cisplatin (100mg/m^2^) every three weeks.

### Tumor Volume Measurement

Three simulation CT scans were performed for every patient: before induction chemotherapy, before radiotherapy and the fourth week of radiotherapy. The primary tumor and the metastatic lymph nodes were delineated on simulation CT images according to the MRI and CT fused images. The volume was automatically measured by Eclipse 10.0 treatment planning system (Varian, CA, USA). The definitions of tumor volume were listed as follows: pre-induction chemotherapy gross primary tumor (preIC GTVnx) and lymph node (preIC GTVnd)、post-induction chemotherapy gross primary tumor(postIC GTVnx) and lymph node (postIC GTVnd), gross primary tumor at fourth week of radiotherapy (midRT GTVnx) and lymph node (midRT GTVnd).

### Evaluation and Statistical Analysis

The follow-up time was calculated from the end of treatment to the last follow-up or death. Patients were regularly evaluated every 3 months during the first two years, every 6 months in the third–fifth years, and then once every year thereafter. The endpoints in this study included overall survival (OS), progression-free survival (PFS) and distant metastasis-free survival (DMFS). OS was defined as the time from end of treatment to death; PFS was measured from the end of treatment to the date of disease progression or death from any causes; DMFS, was defined as the time from end of treatment to first detection of distant metastasis.

The clinical features in different groups were evaluated by the Pearson Chi-square or Fisher’s test. The hazard ratio (HR) of COX proportional regression is used to re-evaluate the prediction of volumetric parameters. Multivariate Cox proportional hazard regression analysis was conducted to explore significant factors associated with OS, PFS and DMFS, and the proportional-hazards assumption was tested with Schoenfeld residuals. Variable risk was expressed as a hazard ratio (HR) with a corresponding 95% confidence interval (95% CI).

Based on the results of multivariable Cox regression analysis, nomogram models were formulated to predict 4-year OS、PFS and DMFS. The performance of the models was evaluated by ROC analysis and calibration curve using 1000 bootstrap resamples based on the training cohort and validation cohort validity. The value of Concordance index (C-index) and the area under the ROC curve (AUC) were used to evaluate the discriminative ability of nomogram, which ranged from 0.5 to 1.0, with 0.5 indicating a random chance while closer to 1.0 indicating a better ability to correctly discriminate the outcome. Decision curve analysis (DCA) was performed in present study as a method for determining the clinical application value of the prediction models by quantifying the net benefit to the patient under different threshold probabilities, and was applied to compare the predictive validity of the nomogram and 8th edition TNM stage in the training cohort and validation cohort (12, 24). Statistical analyses were performed using IBM SPSS Statistics (Version 25.0) and R program (version 3.6.3). The statistical tests were two-sided, and a p-value of < 0.05 was considered statistically significant difference.

## Results

### Patient Characteristics and Survival

The baseline characteristics of 253 LA-NPC patients were listed in **Supplementary Table 1**. There were more men than women (ratio, 2.46:1). The median patient age was 47 years (range:18-70 years). 44.3% (112 of 253) of patients had history of smoking and 29.2% (74 of 253) had history of drinking. Most patients (74.3%) had WHO nonkeratinizing undifferentiated subtype, and the remaining 25.7% of the patients had WHO nonkeratinizing differentiated subtype. Most patients (60.9%) had clinical stage IV disease. EBV DNA copies were detected only in 54 patients (17.8%) using quantitative PCR assay. In total, 73.1% of patients received TP regimen as induction chemotherapy, 22.9% received GP regimen and only 4% received TPF regimen.

At a median follow-up time of 52 months (rang:4-120 months), 66 patients (26.1%) had died, 26 patients (10.3%) experienced locoregional recurrence, 54 patients (21.3%) developed distant metastasis during the follow-up period. The estimated 4-year OS, PFS and DMFS rates were 76.9%, 68.5% and 78.1%, respectively.

### Comparison of Predictive Performance of Tumor Volumetric Parameters

The detailed tumor volumetric parameters were shown in **Supplementary Table 2**. As continuous variables, we quantitatively analyzed and compared the prediction performance of different tumor volumetric parameters for OS, PFS and DMFS. Compared with other parameters, midRT GTVnd was the best predictive factor for OS (HR: 1.043, 95%CI: 1.031-1.055), PFS (HR: 1.040, 95%CI: 1.030- 1.051), and DMFS (HR: 1.046, 95%CI: 1.034 – 1.059) (**Table 1**). For the convenience of subsequent analysis, midRT GTVnd as continuous variables were divided into four groups as follow according to interquartile ranges (IQR): ≤7.85 cm^3^, 7.85-14.70 cm^3^, 14.70-27.50cm^3^ and > 27.50cm^3^.

**Table 1:** Univariate Cox analysis of volumetric parameters in different endpoints.

Parameter	HR (95%CI)		
	OS	PFS	DMFS
preIC GTVnx	1.007 (1.002-1.012)	1.008 (1.003-1.013)	1.005 (0.999-1.011)
postIC GTVnx	1.012 (1.005-1.018)	1.013 (1.007-1.018)	1.008 (1.001-1.015)
midRT GTVnx	1.013 (1.006-1.019)	1.014 (1.008-1.019)	1.009 (1.002-1.016)
preIC GTVnd	1.007 (1.002-1.012)	1.008 (1.003-1.012)	1.009 (1.004-1.014)
postIC GTVnd	1.025 (1.018-1.033)	1.025 (1.019-1.032)	1.028 (1.020-1.036)
midRT GTVnd	1.043 (1.031-1.055)	1.040 (1.030-1.051)	1.046 (1.034-1.059)

HR, hazard ratio; CI, confidence interval; OS, overall survival; PFS, progression-free survival; DMFS, distant metastasis-free survival; preIC GTVnx and preIC GTVnd, pre-induction chemotherapy gross primary tumor and lymph node; postIC GTVnx and postIC GTVnd, post-induction chemotherapy gross primary tumor and lymph node; midRT GTVnx and midRT GTVnd, gross primary tumor at fourth week of radiotherapy and lymph node.

### Nomogram Development

**Figure 1:**
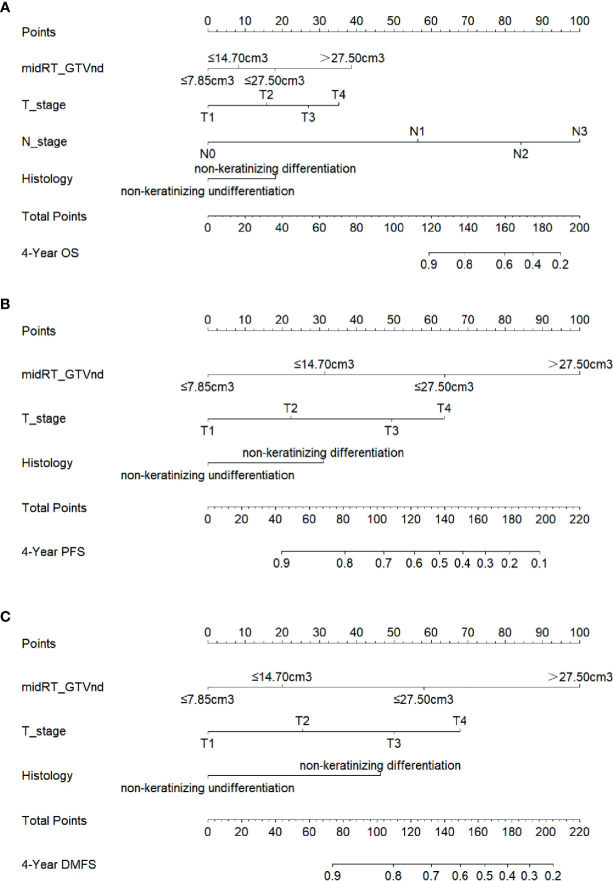
Nomogram to predictive survival. **(A)**, Nomogram for the probability of 4-year OS was developed based on four factors including midRT GTVnd, T stage, N stage and histological type; **(B)**, Nomogram for the probability of 4-year PFS was developed based on three factors including midRT GTVnd, T stage and histological type; **(C)**, Nomogram for the probability of 4-year DMFS was developed based on three factors including midRT GTVnd, T stage, and histological type. The probability could be obtained as function of total points calculated as the sum of points for each specific variable. Points was assigned for each factor by drawing a line upward from the corresponding values to the ‘point’ line. The total sum of points added by each factor was plotted on the “total points” line. A line was drawn down to read the corresponding predictions of probability.

For constructing the nomogram model to predict prognosis of NPC patients received IC followed by CCRT, a total of 253 patients were randomly divided into two independent cohorts according to a 7:3 ratio: training cohort (n = 177) and validation cohort (n =76) (**Table 2**). Univariate and multivariate analysis were conducted to identify prognostic factors associated with survival in the training cohort. The covariates included sex, age, smoking history, drinking history, histological WHO types, T stage, N stage, clinical stage, midRT GTVnd. Based on the multivariate analysis, histological type (P=0.02), T stage (P=0.015), N stage (P=0.027) and midRT GTVnd (P < 0.001) were correlated with OS. For PFS and DMFS, histological type, T stage and midRT GTVnd were detected as independently prognostic factors (P < 0.05) (**Supplementary Tables 3–5**). Based on predictive factors identified from the multivariate analysis in training cohort, we developed three nomograms to predict 4-year OS, PFS and DMFS, respectively (**Figure 1**).

**Table 2:** Characteristics of Patients in the Primary and Validation Cohorts.

Characteristic	Training cohort (n=177)	Validation cohort (n=76)	P value
Sex			0.559
Male	124 (70.1%)	56 (73.7%)	
Female	53 (29.9%)	20 (26.3%)	
Age (years)			
Median age (range)	46.85 (18-70)	47.66 (18-66)	0.555
≤45	72 (40.7%)	30 (39.5%)	0.858
>45	105 (59.3%)	46 (60.5%)	
Smoking			0.922
Yes	78 (44.1%)	34 (44.7%)	
No	99 (55.9%)	42 (55.3%)	
Drinking			0.816
Yes	51 (28.8%)	23 (30.3%)	
No	126 (71.2%)	53 (69.7%)	
Histological type			0.644
non-keratinizing undifferentiation	133 (75.1%)	55 (72.4%)	
non-keratinizing differentiation	44 (24.9%)	21 (27.6%)	
T stage			0.443
T1	12 (6.8%)	3 (3.9%)	
T2	73 (41.2%)	34 (44.7%)	
T3	36 (20.3%)	8 (10.5%)	
T4	56 (31.6%)	31 (40.8%)	
N stage			0.372
N0	1 (0.6%)	0 (0%)	
N1	12 (6.8%)	5 (6.6%)	
N2	100 (56.5%)	49 (64.5%)	
N3	64 (36.2%)	22 (28.9%)	
Disease stage			0.441
III	72 (40.7%)	27 (35.5%)	
IV	105 (59.3%)	49 (64.5%)	
EBV DNA copies			0.820
undetected	147 (83.1%)	64 (84.2%)	
detected	30 (16.9%)	12 (15.8%)	
IC regimens			0.425
TP	127 (71.8%)	58 (76.3%)	
GP	42 (23.7%)	16 (21.1%)	
TPF	8 (4.5%)	2 (2.6%)	
IC cycles			0.442
1 Cycle	1 (0.6%)	2 (2.6%)	
2 Cycles	99 (55.9%)	36 (47.4%)	
3 Cycles	77 (43.5%)	38 (50.0%)	
mid-RT GTVnd			0.201
Median volume (cc) (range)	14.7 (0-99.75)	12.5 (0.1-83.7)	

IC, induction chemotherapy; midRT GTVnd, gross primary tumor at fourth week of radiotherapy.

### Nomogram Validation and Evaluation

**Figure 2:**
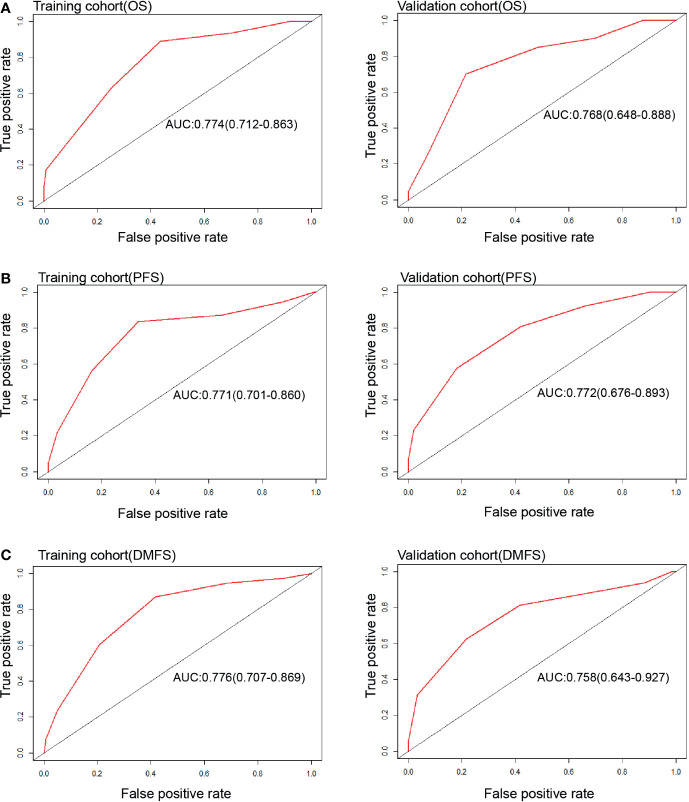
ROC curves of Nomograms to predict 4-year OS **(A)**, PFS **(B)** and DMFS **(C)** in both training and validation cohort.

**Figure 3:**
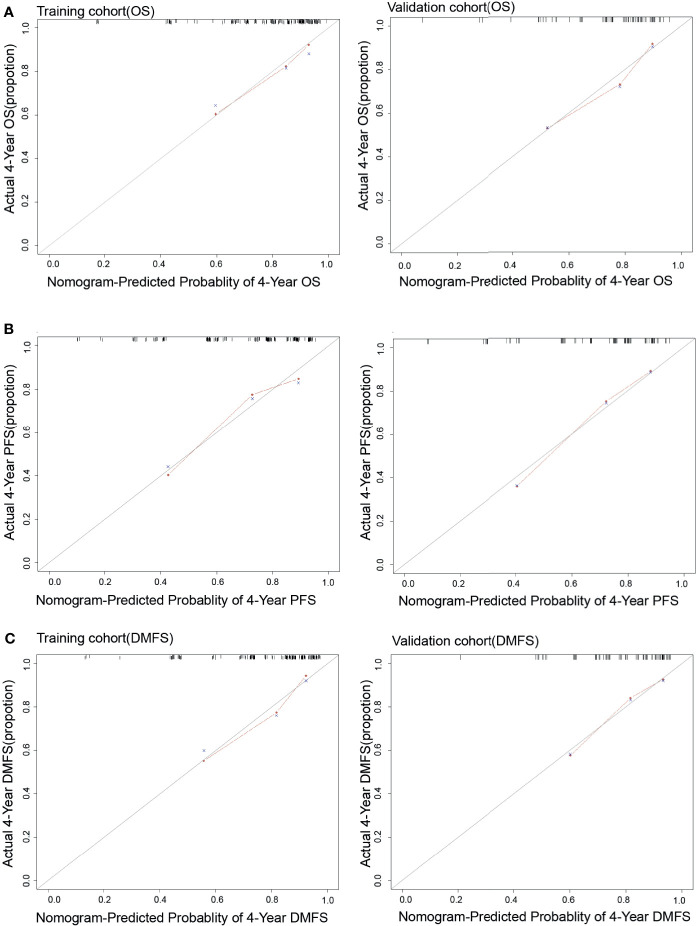
The calibration curves of Nomograms to predict 4-year OS **(A)**, PFS **(B)** and DMFS **(C)** in both training and validation cohort.

**Figure 4:**
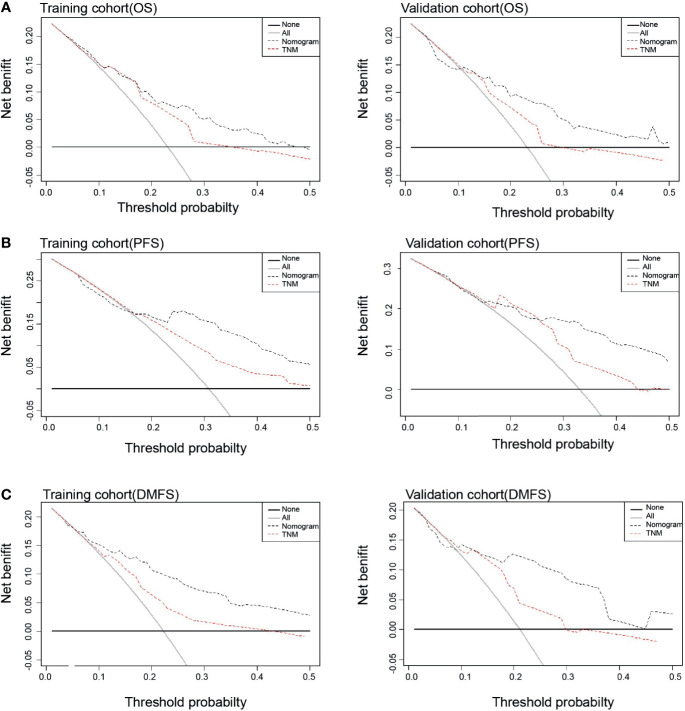
Decision curve analysis of prognostic effects between Nomograms and TNM stage for OS **(A)**, PFS **(B)** and DMFS **(C)** in both training and validation cohort.

Each nomogram was validated internally and externally. The C-index of nomogram to predict OS was 0.746 (95%CI: 0.676-0.816) in training cohort and 0.731 (95%CI: 0.628-0.834) in validation cohort. The AUC showed a good discriminative ability in both cohorts (training cohort, AUC: 0.774, 95%CI 0.712-0.863; validation cohort, AUC: 0.768,95%CI 0.648-0.888). For PFS, The C-index of nomogram was 0.747 (95%CI: 0.684-0.809) in training cohort and 0.735 (95%CI: 0.634-0.836) in validation cohort. And AUC showed a good discriminative ability in both cohorts (training cohort, AUC: 0.771, 95%CI: 0.701-0.860; validation cohort, AUC: 0.772, 95%CI: 0.676-0.893). The C-index of nomogram to predict DMFS was 0.768 (95 CI: 0.699-0.837) in training cohort and 0.729 (95%CI: 0.605-0.852) in validation cohort. The AUC also showed a good discriminative ability in both cohorts (training cohort, AUC: 0.776, 95%CI: 0.707-0.869; validation cohort, AUC: 0.758, 95%CI: 0.643-0.927) (**Figure 2**). Moreover, the calibration plot of each nomogram demonstrated a good consistency between the actual clinical results and the predicted outcomes (**Figure 3**). Then we compared the midRT GTVnd based nomogram against the 8^th^ TNM schema. The DCA showed that the midRT GTVnd based nomogram model was the better reliable clinical tools for predict disease relapse and death (**Figure 4**).

## Discussion

Tumor volume is closely associated with prognosis of NPC has been widely reported (9, 10). Although several studies found pretreatment tumor volume and changing rate of tumor volume before and after IC had prognostic value for NPC, it was not be enough to accurately stratify patients who are benefit from IC followed by CCRT. In this study, we firstly compared the predictive performance of different tumor volumetric parameters in different treatment phase in patients receiving IC followed by CCRT. We found the residual volume of lymph nodes at the fourth week of chemoradiotherapy (midRT GTVnd) had the best predictive effects for OS, PFS and DMFS according to HR of Cox regression analysis, indicating midRT GTVnd was the optimal choice as prognostic factor among all kinds of tumor volumetric parameters in the whole process of IC followed by CCRT. The time point of ART may be a potential factor to impact the predictive effect of midRT GTVnd because tumor volume would be changed along with different ART time point. Although it is still confused to identify the optimal time point of ART, several prospective studies reported change of dose distribution varied markedly at the third or fourth week of radiotherapy in patients with NPC (25, 26). According to these studies, ART is routinely conducted at the fourth week of radiotherapy in our center. Whether other time points of ART could result in different prognostic effects of midRT GTVnd still need to be further investigated for NPC patients.

It has been a consensus that lymph nodes metastasis is associated with poor prognosis of NPC patients. Some specific features of lymph node have also been reported as poor prognostic factors for NPC, such as extracapsular invasion, necrosis, coalescence and bulky disease (>6 cm) which are closely related to the treatment sensitivity (27). In this study, we reported midRT GTVnd was a new feature which could reflect treatment sensitivity because it was defined as the residual volume of lymph nodes after IC plus at least half course of chemoradiotherapy. Analyzing from the potential mechanism, residual volume of lymph nodes might contain large number of treatment resistant cells. It has been confirmed that these cells always contribute to tumor recurrence and metastasis, and further to negatively impact patient survival (28, 29).

On multivariate analysis, we identified histological types, T stage, N stage and midRT GTVnd were independently prognostic factors for OS. In this study, 25.7% of patients had nonkeratinizing differentiated subtype which was associated with poor survival. This result was consistent with our previous studies (20, 30). All patients enrolled in this study were from the Northwest China where were considered as a typical non-endemic area for NPC. Although the prognostic value of histological type for NPC remains controversial in endemic area, given the potentially distinctive pathogenesis, geographical and ethnic origin in Northwest China, the nonkeratinizing differentiated subtype may be an efficient prognostic indicator. We failed to detect a positive correlation between N stage and distant metastatic disease on multivariate analysis. The reason may be explained by unclassified N stage was used to analyze. After patients were divided into two groups: N0-N2 and N3, patients with stage N3 had significantly higher rate of distant metastatic disease than those with stage N0 to N2 using log-rank test (data was not shown).

In view of the prognostic value of midRT GTVnd for OS, PFS and DMFS, we developed and validated three midRT GTVnd based nomograms to predict probability of 4-year survival for LA-NPC patients treated with IC followed by CCRT. The identification and calibration of the nomograms confirmed these prognostic models had wide range of applicability. Compared with the 8^th^ edition of TNM staging system, DCA curves showed the nomogram models had better prediction accuracy for death and disease relapse in patients with LA-NPC receiving IC followed by CCRT. Unlike other risk scores could provide a probability of prognosis before treatment, our models focused on the treatment sensitivity and prognosis at end of the IC followed by CCRT. This would help clinicians to design appropriate strategies of follow-up and adjuvant treatment for each patient.

Although phase 3 trials confirm that adjuvant chemotherapy consist of cisplatin and fluorouracil following chemoradiotherapy fails to yield further benefits in LA-NPC (31, 32), several retrospective studies imply metronomic adjuvant uracil plus tegafur may reduce distant metastasis and improve survival in high-risk patients (33, 34). Plasma Epstein-Barr virus (EBV) DNA of post radiotherapy is often used to guide adjuvant therapy (35). However, different segments of the same viral DNA or different viral genes might result in vary sensitivities in quantitative PCR assay (34). In our center, although plasma EBV DNA is detected routinely using quantitative PCR assay for each patient before treatment and in the whole follow-up period, EBV DNA copies can be detected only in a few plasma samples of patients. Under this situation, these nomogram models established by this study may provide information to stratify high-risk patients without known its plasma EBV DNA status to receive adjuvant chemotherapy. These clinically high-risk features-guided approaches are feasible during daily practice in all hospitals.

The current study may have a few weak points. First, because of its retrospective nature, selection bias might have been unavoidable. Thus, the results need validation of further large sample prospective studies. Second, our data based on a single non-endemic center from the Northwest China, and thus, external validation with other centers in endemic region is needed. Finally, there is a possibility of inter- and/or intra-physician variation in GTV measurements. Despite these limitations, the discriminatory performance of the volumetric parameters in the whole process of IC-CCRT treatment could be utilized as an indicator for tailoring therapy on an individual patient basis.

In this study, we identified the best volumetric factor indicator associated with prognosis was the residual volume of lymph nodes at the fourth week of chemoradiotherapy for NPC patients receiving IC followed by CCRT. Based on the volumetric factor and clinical risk factors, we developed and validated three different nomograms to predict specific probability of 4-year OS, PFS and DMFS for LA-NPC patient, respectively.

## Data Availability Statement

The raw data supporting the conclusions of this article will be made available by the authors, without undue reservation.

## Ethics Statement

The studies involving human participants were reviewed and approved by Medical Ethics Committee of the First Affiliated Hospital of the Fourth Military Medical University. Written informed consent for participation was not required for this study in accordance with the national legislation and the institutional requirements.

## Author Contributions

Study concepts: MS. Study design: YL, JZ, and LZ. Data acquisition: JZ, JL, SL, JW, and BH. Quality control of data and algorithms: YL and MS. Data analysis and interpretation: JZ, JL, YL, LZ, and MS. Statistical analysis: JZ and YL. Manuscript preparation: YL. Manuscript editing: JZ and JL. Manuscript review: MS. All authors contributed to the article and approved the submitted version.

## Conflict of Interest

The authors declare that the research was conducted in the absence of any commercial or financial relationships that could be construed as a potential conflict of interest.

## Publisher’s Note

All claims expressed in this article are solely those of the authors and do not necessarily represent those of their affiliated organizations, or those of the publisher, the editors and the reviewers. Any product that may be evaluated in this article, or claim that may be made by its manufacturer, is not guaranteed or endorsed by the publisher.
