# Supplementary material for: Residual Volume of Lymph Nodes During Chemoradiotherapy Based Nomogram to Predict Survival of Nasopharyngeal Carcinoma Patient Receiving Induction Chemotherapy
Source: Front Oncol. 2021 Sep 6;11:739103. doi: 10.3389/fonc.2021.739103 (PMC8451592; doi:10.3389/fonc.2021.739103)
Supplement: Supplementary file 1 [file Table_1.docx]

Supplementary Material

**Supplementary TABLE 1 |** Clinical Characteristics of all LA-NPC patients

| **Char****acteristic** | **N (%)** |  |
| --- | --- | --- |
| Sex |  |  |
| Male | 180(71.1%) |  |
| Female | 73(28.9%) |  |
| Age(years) |  |  |
| Median age(range) | 47(18-70) |  |
| ≤45 | 102(40.3%) |  |
| >45 | 151(59.7%) |  |
| Smoking |  |  |
| Yes | 112(44.3%) |  |
| No | 141(55.7%) |  |
| Drinking |  |  |
| Yes | 74(29.2%) |  |
| No | 179(70.8%) |  |
| Histological types |  |  |
| nonkeratinizing undifferentiation | 188(74.3%) |  |
| nonkeratinizing differentiation | 65(25.7%) |  |
| T stage |  |  |
| T1 | 15(5.9%) |  |
| T2 | 107(42.3%) |  |
| T3 | 44(17.4%) |  |
| T4 | 87(34.4%) |  |
| N stage |  |  |
| N0 | 1(0.4%) |  |
| N1 | 17(6.7%) |  |
| N2 | 149(58.9%) |  |
| N3 | 86(34%) |  |
| Disease stage |  |  |
| III | 99(39.1%) |  |
| IV | 154(60.9%) |  |
| EBV DNA copies |  |  |
| undetected | 208(82.2%) |  |
| detected | 45(17.8%) |  |
| IC regimens |  |  |
| TP | 185(73.1%) |  |
| GP | 58(22.9%) |  |
| TPF | 10(4%) |  |
| IC cycles |  |  |
| 1 Cycle | 3(1.2%) |  |
| 2 Cycles | 135(53.4%) |  |
| 3 Cycles | 115(45.5%) |  |

IC, induction chemotherapy.

**Supplementary TABLE 2 |** tumor volumetric parameter**s**

| **Time Points** | **Median of volume (range)(cm^3^)** | **reduction rate (%) (range)** |
| --- | --- | --- |
| preIC GTVnx | 38.0(2.0-232.2) |  |
| postIC GTVnx | 29.6(1.5-197.9) |  |
| midRT GTVnx | 28.4(2.0-188.0) |  |
| preIC GTVnd | 42.6(0.0-256.0) |  |
| postIC GTVnd | 20.5(0.0-148.3) |  |
| midRT GTVnd | 13.9(0.0-99.7) |  |
| PVRR-IC |  | 15.66(-50 to 87) |
| PVRR-POM |  | 1.78(-69-62) |
| PVRR-PEM |  | 21.11(-51-85) |
| LVRR-IC |  | 40.44(-225-94) |
| LVRR-POM |  | 21.35(-55-98) |
| LVRR-PEM |  | 63.08(-156-99) |

preIC GTVnx and preIC GTVnd, pre-induction chemotherapy gross primary tumor and lymph node; postIC GTVnx and postIC GTVnd, post-induction chemotherapy gross primary tumor and lymph node; midRT GTVnx and midRT GTVnd, gross primary tumor at fourth week of radiotherapy and lymph node; PVRR-IC, primary tumor volume reduction rate before and after IC; LVRR-IC, lymph node volume reduction rate before and after IC; PVRR-POM, primary tumor volume reduction rate from post IC to midRT; LVRR-POM, lymph node volume reduction rate from post IC to midRT; PVRR-PEM, primary tumor volume reduction rate from preIC to midRT; LVRR-PEM, lymph node volume reduction rate from preIC to midRT.

**Supplementary TABLE 3 |** Univariate and multivariate Cox analysis of OS in the whole cohort

| **Variable** | **Univariate analysis**  **HR (95% CI) P value** | | **Multivariate analysis**  **HR (95% CI) P value** | | |
| --- | --- | --- | --- | --- | --- |
| Sex | 1.385(0.701-2.734) | 0.348 |  |  |  |
| Female |  |  |  |  |  |
| Male |  |  |  |  |  |
| Age | 1.246(0.684-2.270) | 0.472 |  |  |  |
| ≤45 |  |  |  |  |  |
| ＞45 |  |  |  |  |  |
| Smoking | 1.125(0.628-2.015) | 0.693 |  |  |  |
| No |  |  |  |  |  |
| Yes |  |  |  |  |  |
| Drinking | 1.074(0.573-2.012) | 0.823 |  |  |  |
| No |  |  |  |  |  |
| Yes |  |  |  |  |  |
| Histological type | 2.269(1.261-4.084) | 0.006 | 2.074(1.124-3.828) | 0.020 |  |
| nonkeratinizing undifferentiation |  |  |  |  |  |
| nonkeratinizing differentiation |  |  |  |  |  |
| T stage | 1.304(0.960-1.772) | 0.089 | 1.478(1.083-2.017) | 0.014 |  |
| T1 |  |  |  |  |  |
| T2 |  |  |  |  |  |
| T3 |  |  |  |  |  |
| T4 |  |  |  |  |  |
| N stage | 2.368(1.391-4.034) | 0.002 | 1.898(1.075-3.349) | 0.027 |  |
| N0 |  |  |  |  |  |
| N1 |  |  |  |  |  |
| N2 |  |  |  |  |  |
| N3 |  |  |  |  |  |
| EBV DNA copies | 0.683(0.269-1.735) | 0.423 |  |  |  |
| undetected |  |  |  |  |  |
| detected |  |  |  |  |  |
| mid-RT GTVnd | 1.797(1.339-2.413) | <0.001 | 1.722(1.278-2.321) | <0.001 |  |
| ≤7.85 |  |  |  |  |  |
| 7.85-14.70 |  |  |  |  |  |
| 14.70-27.50 |  |  |  |  |  |
| >27.50 |  |  |  |  |  |

HR, hazard ratio; CI, confidence interval; OS, overall survival; midRT GTVnd, gross primary tumor at fourth week of radiotherapy

**Supplementary TABLE 4 |** Univariate and multivariate Cox analysis of PFS in the whole cohort

| **Variable** | **Univariate analysis**  **HR (95% CI) P value** | | **Multivariate analysis**  **HR (95% CI) P value** | | |
| --- | --- | --- | --- | --- | --- |
| Sex | 1.604(0.846-3.043) | 0.148 |  |  |  |
| Female |  |  |  |  |  |
| Male |  |  |  |  |  |
| Age | 1.226(0.707-2.124) | 0.468 |  |  |  |
| ≤45 |  |  |  |  |  |
| ＞45 |  |  |  |  |  |
| Smoking | 1.872(1.095-3.200) | 0.022 | 1.264(0.647-2.470) | 0.493 |  |
| No |  |  |  |  |  |
| Yes |  |  |  |  |  |
| Drinking | 1.645(0.949-2850) | 0.076 |  |  |  |
| No |  |  |  |  |  |
| Yes |  |  |  |  |  |
| Histological type | 1.886(1.094-3.250) | 0.022 | 1.809(1.030-3.179) | 0.039 |  |
| non-keratinizing undifferentiation |  |  |  |  |  |
| non-keratinizing differentiation |  |  |  |  |  |
| T stage | 1.452(1.095-1.924) | 0.010 | 1.564(1.169-2.093) | 0.003 |  |
| T1 |  |  |  |  |  |
| T2 |  |  |  |  |  |
| T3 |  |  |  |  |  |
| T4 |  |  |  |  |  |
| N stage | 1.558(0.996-2.435) | 0.052 | 1.368(0.854-2.193) | 0.193 |  |
| N0 |  |  |  |  |  |
| N1 |  |  |  |  |  |
| N2 |  |  |  |  |  |
| N3 |  |  |  |  |  |
| EBV DNA copies | 0.836(0.394-1.771) | 0.836 |  |  |  |
| undetected |  |  |  |  |  |
| detected |  |  |  |  |  |
| mid-RT GTVnd | 1.858(1.435-2.407) | <0.001 | 1.842(1.404-2.417) | <0.001 |  |
| ≤7.85 |  |  |  |  |  |
| 7.85-14.70 |  |  |  |  |  |
| 14.70-27.50 |  |  |  |  |  |
| >27.50 |  |  |  |  |  |

HR, hazard ratio; CI, confidence interval; PFS, progression-free survival; midRT GTVnd, gross primary tumor at fourth week of radiotherapy.

**Supplementary TABLE 5 |** Univariate and multivariate Cox analysis of DMFS in the whole cohort

| Variable | Univariate analysis  HR (95% CI) P value | | Multivariate analysis  HR (95% CI) P value | | |
| --- | --- | --- | --- | --- | --- |
| Sex | 1.468(0.695-3.102) | 0.314 |  |  |  |
| Female |  |  |  |  |  |
| Male |  |  |  |  |  |
| Age | 1.173(0.600-2.292) | 0.642 |  |  |  |
| ≤45 |  |  |  |  |  |
| ＞45 |  |  |  |  |  |
| Smoking | 1.684(0.888-3.193) | 0.110 |  |  |  |
| No |  |  |  |  |  |
| Yes |  |  |  |  |  |
| Drinking | 1.908(1.002-3.634) | 0.049 | 2.072(0.901-4.767) | 0.086 |  |
| No |  |  |  |  |  |
| Yes |  |  |  |  |  |
| Histological type | 2.293(1.209-4.347) | 0.011 | 2.551(1.333-4.880) | 0.005 |  |
| nonkeratinizing undifferentiation |  |  |  |  |  |
| nonkeratinizing differentiation |  |  |  |  |  |
| T stage | 1.441(1.031-2.012) | 0.032 | 1.615(1.149-2.271) | <0.006 |  |
| T1 |  |  |  |  |  |
| T2 |  |  |  |  |  |
| T3 |  |  |  |  |  |
| T4 |  |  |  |  |  |
| N stage | 1.633(0.918-2.906) | 0.095 |  |  |  |
| N0 |  |  |  |  |  |
| N1 |  |  |  |  |  |
| N2 |  |  |  |  |  |
| N3 |  |  |  |  |  |
| EBV DNA copies | 0.744(0.291-1.906) | 0.538 |  |  |  |
| undetected |  |  |  |  |  |
| detected |  |  |  |  |  |
| mid-RT GTVnd | 2.035(1.460-2.837) | <0.001 | 2.033(1.440-2.871) | <0.001 |  |
| ≤7.85 |  |  |  |  |  |
| 7.85-14.70 |  |  |  |  |  |
| 14.70-27.50 |  |  |  |  |  |
| >27.50 |  |  |  |  |  |

HR, hazard ratio; CI, confidence interval; DMFS, distant metastasis-free survival; midRT GTVnd, gross primary tumor at fourth week of radiotherapy.
